# Evaluation of bactericidal and anti-biofilm properties of a novel surface-active organosilane biocide against healthcare associated pathogens and *Pseudomonas aeruginosa* biolfilm

**DOI:** 10.1371/journal.pone.0182624

**Published:** 2017-08-07

**Authors:** Jason Murray, Tendai Muruko, Chris I. R. Gill, M. Patricia Kearney, David Farren, Michael G. Scott, Geoff McMullan, Nigel G. Ternan

**Affiliations:** 1 Nutrition Innovation Centre for food and HEalth (NICHE), School of Biomedical Sciences, University of Ulster, Coleraine, Co. Londonderry, Northern Ireland, United Kingdom; 2 Northern Health and Social Care Trust, Antrim area Hospital, Bush House, Antrim, Co. Antrim, Northern Ireland, United Kingdom; 3 Institute for Global Food Security, School of Biological Sciences, Medical Biology Centre, Queens University Belfast, Belfast, Northern Ireland, United Kingdom; University of Connecticut, UNITED STATES

## Abstract

Healthcare acquired infections (HAI) pose a great threat in hospital settings and environmental contamination can be attributed to the spread of these. De-contamination and, significantly, prevention of re-contamination of the environment could help in preventing/reducing this threat. Goldshield (GS5) is a novel organosilane biocide marketed as a single application product with residual biocidal activity. We tested the hypothesis that GS5 could provide longer-term residual antimicrobial activity than existing disinfectants once applied to surfaces. Thus, the residual bactericidal properties of GS5, Actichlor and Distel against repeated challenge with *Staphylococcus aureus* ATCC43300 were tested, and showed that GS5 alone exhibited longer-term bactericidal activity for up to 6 days on 316I stainless steel surfaces. Having established efficacy against *S*. *aureus*, we tested GS5 against common healthcare acquired pathogens, and demonstrated that, on average, a 1 log^10^ bactericidal effect was exhibited by GS5 treated surfaces, although biocidal activity varied depending upon the surface type and the species of bacteria. The ability of GS5 to prevent *Pseudomonas aeruginosa* biofilm formation was measured in standard microtitre plate assays, where it had no significant effect on either biofilm formation or development. Taken together the data suggests that GS5 treatment of surfaces may be a useful means to reducing bacterial contamination in the context of infection control practices.

## Introduction

Healthcare acquired infections (HAIs) are directly and indirectly responsible for increased morbidity and mortality rates in hospitals worldwide. In Europe alone there are >4.5 million cases annually, which result in >37,000 deaths [1]. A further consequence is the financial burden associated with these infections, measured in terms of increased length of patient stay, decreased bed availability as a result and the extra cost of antibiotic therapy to treat the infection. In the USA alone the total annual expenditure on HAI is estimated to be in excess of $9.8 billion (£6–7 billion) [2], while in Europe a figure of over €7 billion (~£5.5 billion) has been proposed [3]. As a consequence, there is increasing interest from industrial, research and development and healthcare sectors in the development of viable and cost-effective alternative methods of reducing HAI.

Common healthcare associated pathogens include *Staphylococcus aureus* (and predominantly Methicillin resistant *Staphylococcus aureus* (MRSA)), Vancomycin-resistant *Enterococci* (VRE), *Clostridium difficile*, and *Pseudomonas aeruginosa*. Such microorganisms have been shown to survive on inanimate surfaces for extended periods of time—for example *S*. *aureus* has been shown to survive as long as 6 months [4,5] while *Enterococci* can survive as long as 4 months [6]. *Clostridium difficile* infections (CDI), the most common HAI type in Europe [7] are attributed in part to the persistence of infectious spores on hospital surfaces for up to 5 months [5]. Bacteria capable of forming biofilms, such as *P*. *aeruginosa* and *S*. *aureus*, also survive and persist in the environment due to this ability, on top of any intrinsic resistance to antimicrobials [8]. Thus vegetative cells, spores, or biofilms present a threat of infection and indeed a recent report identified biofilm within water taps as the cause of a series of neonatal *P*. *aeruginosa* infections [9].

Evidence of a direct correlation between environmental contamination and infection rates exists [5,10,11,12] and microbial contamination of the environment has been shown to act as a source of infection that is directly responsible for transmission of organisms to patients [12]. The most problematic areas tend to be high-touch points such as bed rails, door handles, table top surfaces, bedding (mattress), television controls and staff uniforms [13]. Such contaminated surfaces act as a source of direct to patient, and indirect—via healthcare workers/instruments—spread to patients [5,14]. As long as these organisms persist in a hospital or healthcare facility environment they remain a source of infection and therefore, hospitals have implemented revised and improved infection control practices in order to reduce and ideally eradicate environmental microbial contamination. This is achieved primarily by the use of disinfectants and detergents, although the precise disinfectant used will be dependent on multiple factors. For example, areas of high risk such as operating theatres will require multiple cleans per day, whereas patient waiting rooms may be cleaned only once per day. The choice of disinfectant agent is also multifactorial: body fluid spillages will normally require higher level disinfectants than those used in routine cleaning. As a result, hospitals will use a variety of products including ethyl alcohol in hand rubs and gels, Quaternary ammonium compounds (QACs), chlorine-releasing agents and peroxygen sterilants [15]. Nonetheless, current cleaning methods have in several instances been shown to be ineffective. Work by French *et al*. [11] showed that 74% of sites in a London hospital were MRSA positive and when these same sites were retested post-cleaning, all were still contaminated [11]. Recurrence of contamination on surfaces, post disinfection, is therefore a significant issue and this is especially true of high-touch surfaces [16]. Given the available evidence for the ineffectiveness of cleaning and rapid recontamination of surfaces, there is currently much interest in alternative approaches to the problem. The development of intrinsically anti-microbial surfaces that incorporate a variety of agents to kill microbes may be considered a useful strategy. Alternatively, the use of specialised agents that are capable of preventing surface contamination, or that exhibit a residual antimicrobial activity post-disinfection, could be employed, and such products have recently been highlighted as of potential utility in the healthcare setting [17].

One such antimicrobial product is Goldshield, distributed by Goldshield Technologies Ltd. [GS hereinafter]. This is a patented, water soluble organosilane, coupled with a quaternary ammonium compound that is designed to coat surfaces with a protective antimicrobial layer to prevent microbial contamination. The product was originally designed at Emory University, USA and is the subject of three US patents (patent nos. US5,959,014, US6,221,944, and US6,632,805). In this paper we report the bactericidal and anti-biofilm of GS5 technology against 11 common healthcare associated pathogens.

## Materials and methods

### Chemicals, glassware and media

All glassware was sterilised by soaking overnight in 1% Virkon (Antec, UK) and steam sterilised in an autoclave prior to use. All culture media (Oxoid, UK) was prepared as per the manufacturer’s instructions. Phosphate Buffered Saline (Oxoid, UK) was prepared in deionised water and steam sterilised in an autoclave prior to use. Two model surfaces were used. 316l Steel (Aalco, UK) or Formica were cut into 2cm×2cm samples, autoclaved (121°C for 15 min) and stored in a sealed sterile container prior to use.

### Microorganisms

Ten bacterial species were obtained from either the American Type Culture Collection (ATCC) or the Leibniz-Institute DSMZ German Collection of Microorganisms and Cell Cultures (DSMZ). Bacteria included *Escherichia coli* ATCC25922, *Klebsiella pneumoniae* DSM16358, *Mycobacterium smegmatis* DSM43469, *Pseudomonas aeruginosa* DSM3227, *Staphylococcus aureus* (MRSA) ATCC43300, *Staphylococcus aureus* (non-MRSA) DSM20231, *Staphylococcus epidermidis* DSM28319 (all cultured at 37°C using Nutrient broth/agar), *Enterococcus faecalis* DSM12956 (37°C using Tryptone soya broth/agar), *Burkholderia multivorans* DSM13243 (28°C using Nutrient broth/agar) and *Acinetobacter baumannii* DSM30008 (30°C using Nutrient broth and agar). These were chosen as representative organisms of the type causing HAIs commonly seen in hospitals [18] and included Gram positive organisms, Gram negative organisms and *Mycobacteria*. *Mycobacterium smegmatis* was used as it is a fasting-growing model *Mycobacterium* species [19]. Organisms were stored on Cryobeads (Technical Service Consultants Ltd, UK) at -80°C and recovered in suitable media when required.

### Disinfectant agents

Three disinfectant agents used (GS5, Actichlor and Distel) are classed bactericidal surface disinfectants. The characteristics of these antimicrobial agents are summarised in Table 1. Agents were acquired as full strength concentrate and working stock concentrations were prepared by dilution with deionised water as per the respective manufacturer’s instructions.

**Table 1:** Antimicrobial products tested.

Agent	Type	Active ingredient	Concentration used*
**Goldshield5**	Organosilane coupled with Quaternary Ammonium Compound (siQAC)	Trihydroxysilylpropyldimethyloctadecyl ammonium chloride	1:20 dilution
**Actichlor**	Chlorine-based disinfectant	Sodium dichloroisocyanurate	1:10 dilution
**Distel**	Quaternary Ammonium Compound	Tertiary alylamine and quaternary ammonium compounds	1:100 dilution

* as per manufacturer’s instructions.

### Direct bactericidal assessment of GS5

To determine directly the bactericidal activity of GS5, a suspension contact time assay was completed; varying concentrations of GS5 were mixed with *S*. *aureus* ATCC43300, followed by recovery and enumeration of viable cells to determine Log_10_ reduction. 0% (sterile water), 0.25% (v/v), 0.5% (v/v) and 1% (v/v) GS5 dilutions were prepared using sterile water as diluent. A 10 μl aliquot of mid-log *S*. *aureus* ATCC43300 was mixed with each of the GS5 concentrations and left to stand at room temperature for 5 min. Bacteria were enumerated by dilution plating 0.1ml aliquots onto Nutrient agar in duplicates and incubating at 37°C for 24 h followed by direct colony counts. Three biologically independent experiments were performed.

### Residual surface activity of disinfectants

To investigate the residual activity of surface disinfectants a protocol was developed from the EN13697 standard and the work of Baxa *et al*. [20]. *Staphylococcus aureus* ATCC43300 (MRSA) and 316l Steel were used. The 316l Steel surface samples were sprayed with either GS5, Actichlor, Distel or sterile water (no treatment control) using a hand spray. The test surfaces were left to dry in the sterile environment of a category 2 cabinet (Biomat). *S*. *aureus* ATCC43300 was grown to mid log phase of growth (OD_600_ = ~0.48) and diluted 1/100 using sterile PBS (Oxoid, UK). A total of 100 μl of this was added (in 10 μl droplets) to technical triplicate examples of each surface. Bacteria were left on the surfaces for 45 min, and then viable cells recovered in 10 ml of sterile PBS by vortexing for 2 min. Bacteria were enumerated by plating dilution series in duplicate on Nutrient Agar and incubating at 37°C for 24 h followed by direct colony counts [20]. Following recovery of bacteria from the surfaces each surface was individually washed using sterile PBS, air dried and stored in a sterile storage box. These surfaces were then re-challenged with *S*. *aureus* ATCC43300 as above. This re-challenge was repeated at 3-day intervals over 15 days. Three biologically independent experiments were performed.

### GS5 bactericidal surface testing

A selection of 10 different bacteria, representative of important HAI, were individually tested on 316l Steel and Formica. Testing was performed once to determine the maximum antimicrobial effect for a freshly treated surface. The protocol was as described above, but without re-challenge and only the activity of GS5 was assessed.

### Assessment of GS5 efficacy against biofilms

*Pseudomonas aeruginosa* DSM3227 biofilms were grown in 24-well microtiter plates (4 wells per treatment) and these were stained with 0.1% crystal violet to assess the extent of biofilm growth according to established methods [21,22,23]. To determine efficacy of GS5 against biofilm, Thermo Scientific^™^ Nunc^™^ Cell-Culture Treated Multidishes, (Thermo Scientific, UK) were pre-treated with either 5% GS5 or sterile water (untreated): wells were soaked with 1 ml of agent for 10 min following which treatment agents were aspirated and plates left to dry in a sterile environment (Biomat category 2 cabinet). An overnight culture of *P*. *aeruginosa* DSM3227 was diluted 1/100 (using sterile nutrient broth) and microtitre plate wells inoculated with a 1 ml aliquot following which the plates were incubated aerobically at 37°C. At defined time points (8 h, 12 h, 24 h, 48 h, 72 h and 96 h) biofilm production was assessed. The medium containing planktonic cells was removed and wells stained with 1.5 ml of 0.1% Crystal Violet (Sigma-Aldrich, UK) for 10 min at room temperature. Unbound crystal violet (Sigma-Aldrich, UK) was removed and stained wells washed twice with 2ml sterile PBS following which bound crystal violet was solubilised using 1.5 ml of 30% Acetic Acid (Thermo Scientific, UK) for 30 min at room temperature. A 1 ml aliquot from each well was transferred to a fresh 24-well microtiter plate and the absorbance of the crystal violet measured at 570nm using a FLUROstar Omega plate reader (BMG LABTECH, Europe). Each experiment was repeated on three separate occasions.

### Assessment of GS5 effects on bacterial viability in biofilm

Bacterial viability in biofilms was assessed using the BacLight Live/Dead bacterial viability kit (L-7007; Molecular Probes, Eugene, OR) [24,25]. With Baclight, live cells stain green and dead/damaged cells stain red. A stock solution was prepared by mixing 4 μl of component A (1.67 mM Syto9 plus 1.67 mM propidium iodide), 6 μl of component B (1.67 mM syto9 plus 18.3 mM propidium iodide) and 1ml of sterile water as described by Bauer *et al*. [25]. *P*. *aeruginosa* DSM3227 biofilm was grown in 4-well Nunc^™^ Lab-Tek^™^ II Chamber Slide^™^ Systems (Thermo Scientific, UK) pre-treated with either 5% GS5 or sterile deionised water. Slides were inoculated with 1 ml of a 1/100 dilution of overnight culture of *P*. *aeruginosa* and incubated aerobically for 24 h and 48 h at 37°C. At each time point excess media and planktonic cells were removed and the wells washed with sterile PBS followed by staining with 200 μl BacLight mix and 100 μl of sterile water. Stained slides were incubated in the dark at room temperature for 30 min following which the wells were then washed with sterile PBS and viewed using ×100 oil immersion on a Nikon ECLIPSE E400 (Nikon) microscope utilising a dual-band emission filter (450–490 nm/510–560 nm). Images were generated using NIS-Elements BR (Nikon) software version 3.22.09. Image J software was used to generate composite (red/green) images of the baclight stained biofilms.

### Statistical analysis

For bactericidal testing, log_10_ changes in viable bacterial numbers, compared to untreated controls was determined. The equation Log Reduction LR = log_10_ (N_control_)–log10 (N_treated_) was used where *N*_*control*_ is total recovery of untreated bacteria and *N*_*treated*_ is total recovery of treated bacteria. Data was imported to Graphpad Prism 6.01 and charts constructed. Statistical analysis was completed using SPSS v22.

## Results

### Direct bactericidal assessment of GS5

**Fig 1:**
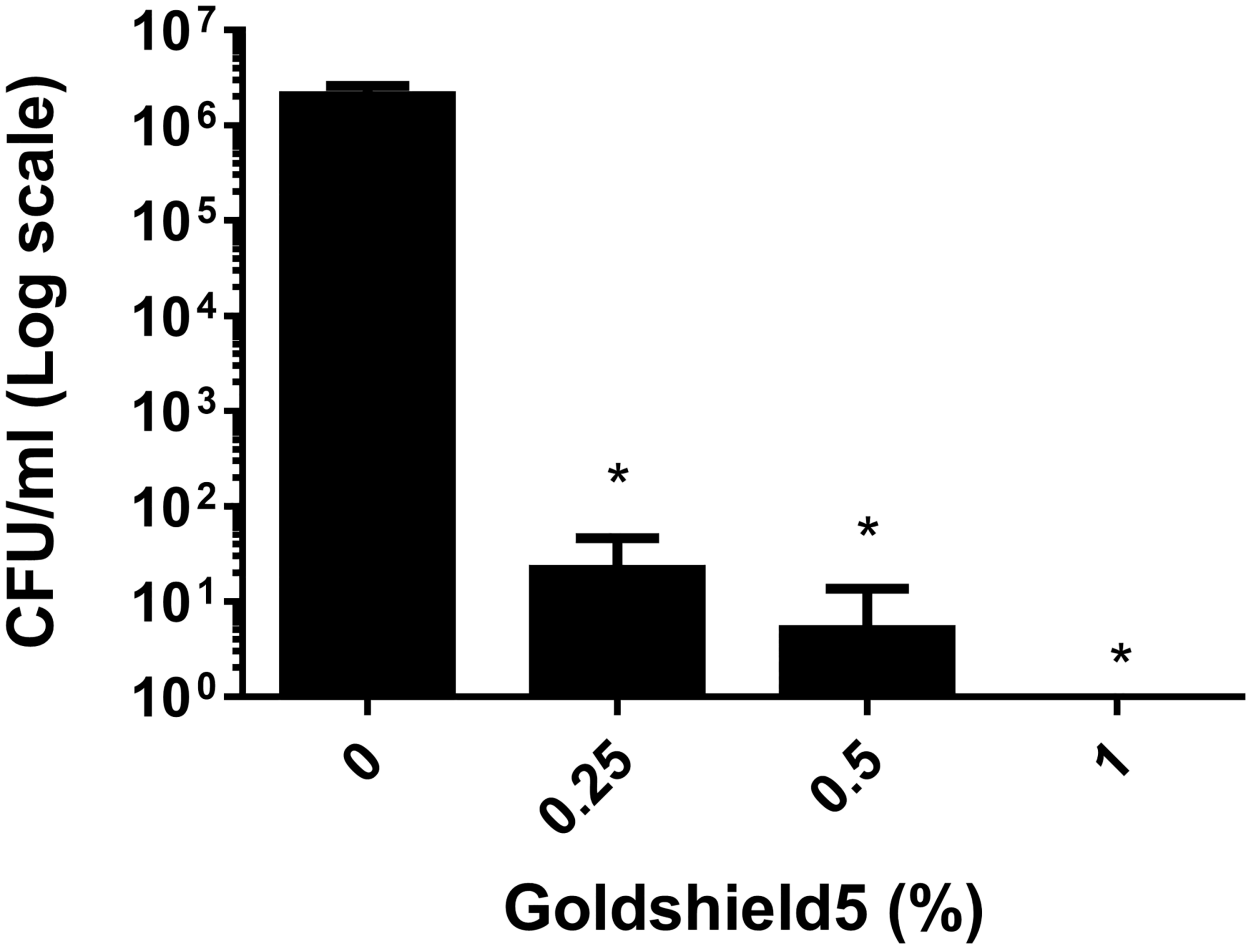
*S*. *aureus* ATCC43300 survival following suspension test using GS5. ~2×10^6^ cfu/ml of *S*. *aureus* ATCC43300 was challenged with increasing concentrations of GS5. Data represents mean +/- SD of three independent experiments. Statistical analysis by independent T-tests versus Untreated (0%) controls (* = p<0.05, ** = p<0.005, *** = p<0.001).

We firstly wished to determine if GS5 was effective against bacteria in solution, prior to surface testing. We hypothesised that a solution of GS5 at working concentration would exhibit a bactericidal effect against a suspension of bacteria. The direct antibacterial effects of GS5 against *S*. *aureus* ATCC43300 was assessed using a suspension assay. *S*. *aureus* ATCC43300 was challenged with increasing concentrations of GS5 to quantify bactericidal activity. GS5 exhibited bactericidal actions at all concentrations after 5min contact time as shown in Fig 1 (0.25% = 4.96 Log_10_ reduction; 0.5% = 5.6 Log_10_ reduction; 1% = 6 Log_10_ reduction (complete kill). Subsequent testing was completed at 5% as per manufacturer’s instructions.

### Residual activity of surface disinfectants

**Fig 2:**
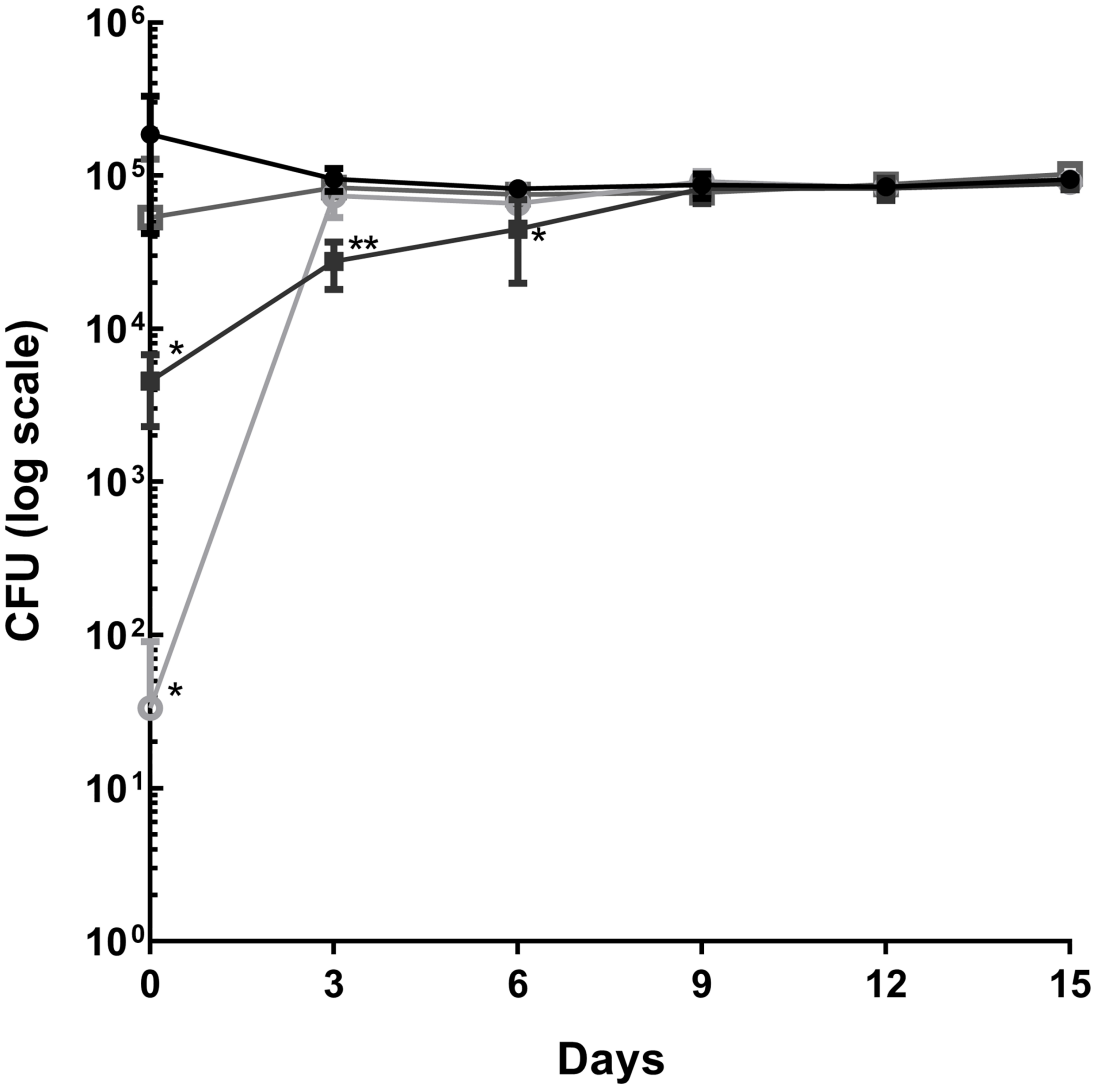
Comparison of residual antimicrobial effects of GS5, Actichlor and Distel on steel surface loaded with *Staphylococcus aureus* ATCC43300. GS5 exhibited prolonged antibacterial activity (6 days) whereas Actichlor and Distel showed no antibacterial activity after day 0. Results are representative of three independent experiments (n = 3; mean+/- SD plotted). Statistical analysis using One way ANOVA and Dunnett’s T-test versus Untreated control (* = p<0.05, ** = p<0.005, *** = p<0.001). ▪ = Goldshield; ● = Untreated control; ○ = Actichlor; □ = Distel.

GS5 is reported to form covalent bonds with surfaces, thereby leaving a nanoscale antimicrobial coating which kills microbes that encounter that surface. This, it is claimed, makes GS5 a more effective product due to its residual antimicrobial activity compared to other disinfectants. We designed an experiment to test this hypothesis by determining the residual antimicrobial effect of GS5, Actichlor and Distel. The bactericidal activity of the three surface disinfectant agents was tested against *S*. *aureus* ATCC43300 on 316l Steel (Aalco, UK) and residual activity was assessed over 15 days at 3 day intervals. All three products exhibit bactericidal activity on day 0 (Actichlor = 3.75Log_10_ reduction; Distel = 0.54 Log_10_reduction; GS5 = 1.16 Log_10_ reduction). Following subsequent re-challenge of treated surfaces with *S*. *aureus* ATCC43300 only GS5 showed significant residual bactericidal activity; this residual activity exerted by GS5 was evident for 6 days (Day 3 GS5 = 0.53 Log_10_ reduction; Day 6 GS5 = 0.26 Log_10_ reduction; Fig 2). For subsequent testing of the GS5 product, the maximum effect time point (day 0) was used.

### GS5 bactericidal surface testing

Baxa *et al*. [20] suggested that GS5 exhibited variable effect against different bacterial species. We therefore tested GS5 against a range of healthcare acquired infection microorganisms on 316l Steel or Formica to determine bactericidal effect. As hypothesised, GS5 treated surfaces did indeed exhibit a bactericidal effect against all ten tested microorganisms, and this effect was observed on both Formica and steel. The largest bactericidal effect was observed with *Staphylococcus* strains where a >1 log_10_ reduction was observed on 316l Steel (*S*. *aureus* ATCC43300 = 1.21 Log_10_ reduction; *S*. *epidermidis* DSM28319 = 1.06 Log_10_reduction) (Table 2). On Formica, however, the GS5 product exhibited a lower bactericidal effect (<0.5 = Log_10_reduction) against both *Staphylococcus* organisms. The average Log_10_ reduction on steel surfaces for all bacteria tested was 0.6, whereas the average Log_10_ reduction on Formica was 0.45.

**Table 2:** Log_10_ reductions obtained on GS5 treated surfaces challenged with a variety of microbes.

Organism	Surface	Log_10_ Untreated ± SD	Log_10_ Treated ± SD	Log_10_ change	*p-value*
***Acinetobacter baumannii* DSM30008**	Steel	4.82 ±0.36	4.49 ±0.62	0.33*	**0.0138**
	Formica	4.25 ±0.04	3.67 ±0.29	0.58***	**<0.001**
***Burkholderia multivorans* DSM13243**	Steel	3.90 ±0.14	3.62 ±0.17	0.28***	**<0.001**
	Formica	3.94 ±0.05	3.41 ±0.24	0.53**	**0.0011**
***Enterococcus faecalis* DSM12956**	Steel	5.27 ±0.3	4.8 ±0.08	**0.47**	**0.0623**
	Formica	5.15 ±0.13	4.86 ±0.03	0.29**	**0.0016**
***Escherichia coli* ATCC25922**	Steel	5.57±0.28	5.32 ±0.33	0.25**	**0.0018**
	Formica	5.54 ±0.09	5.22 ±0.02	0.32***	**<0.001**
***Klebsiella pneumoniae* DSM16358**	Steel	4.30±0.27	3.54 ±0.33	0.76*	**0.0135**
	Formica	3.94 ±0.05	3.41 ±0.24	0.53**	**0.0011**
***Mycobacterium smegmatis* DSM43469**	Steel	4.06 ±0.22	3.46 ±0.45	0.6***	**<0.001**
	Formica	5.83 ±0.43	5.16 ±0.44	0.67**	**0.0026**
***Pseudomonas aeruginosa* DSM3227**	Steel	5.09±0.04	4.66±0.29	0.43**	**0.0017**
	Formica	5.15±0.1	4.63±0.12	0.52***	**<0.001**
***Staphylococcus aureus* (MRSA) ATCC43300**	Steel	4.19 ±0.13	2.99 ±0.58	1.2***	**<0.001**
	Formica	5.04 ±0.03	4.68 ±0.08	0.36***	**<0.001**
***Staphylococcus aureus* (non-MRSA) DSM20231**	Steel	4.57±0.22	3.48±0.27	1.09***	**<0.001**
	Formica	5.02±0.23	3.94±0.35	1.08*	**0.0089**
***Staphylococcus epidermidis* DSM28319**	Steel	3.95 ±0.04	2.88 ±0.05	1.07**	**0.0047**
	Formica	5.25 ±0.19	4.94 ±0.25	0.31***	**<0.001**

Results are representative of three independent experiments (n = 3; mean+/- SD). *p* value calculated using paired T-Test (* = p<0.05, ** = p<0.005, *** = p<0.001).

### Effect of GS5 on bacterial biofilm formation

**Fig 3:**
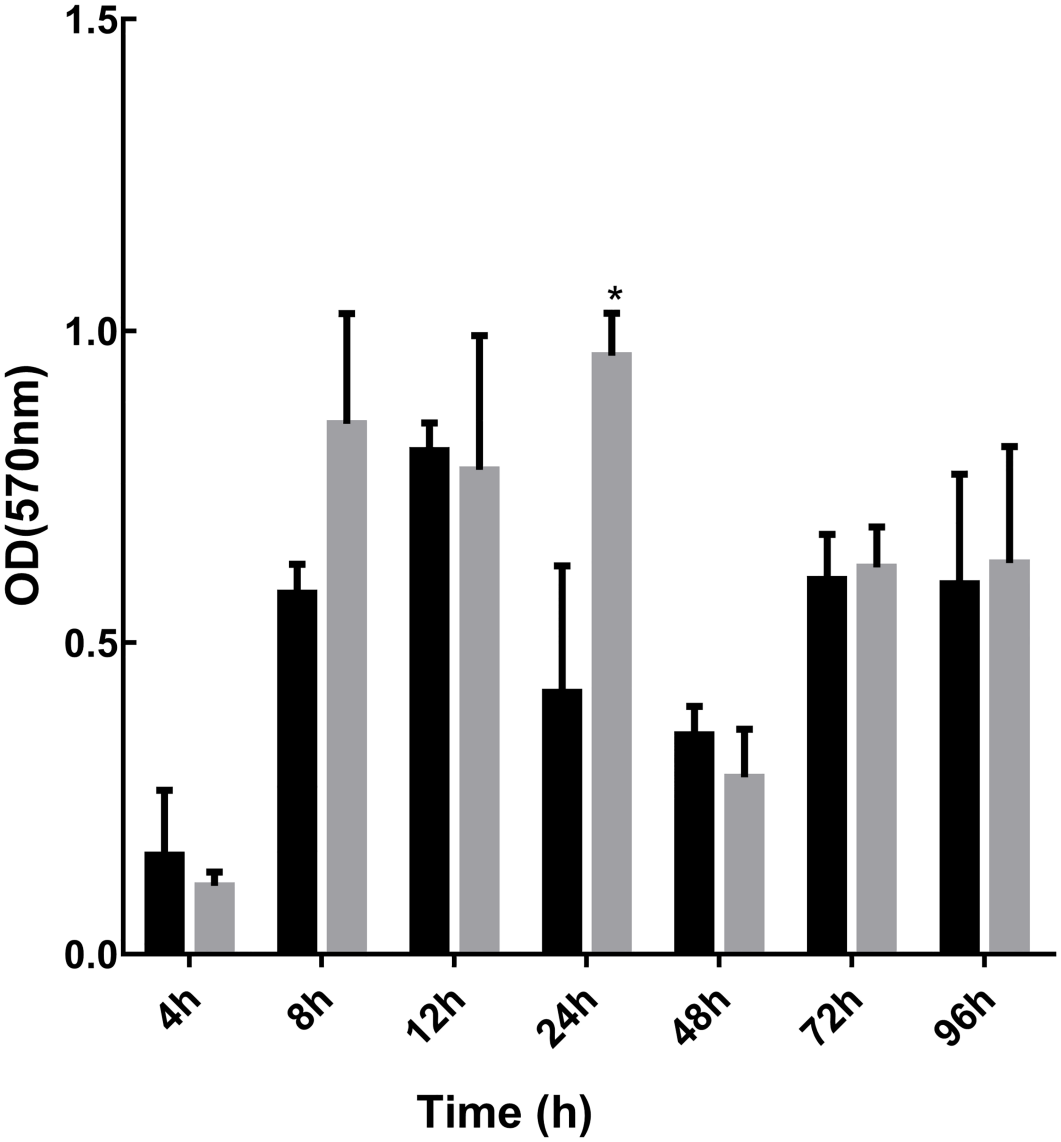
Biofilm development following pre-treatment with GS5. *Pseudomonas aeruginosa* DSM3227 biofilm biomass was assessed by crystal violet staining at various time points and data presented represents mean +/- SD of three independent experiments. Statistical analysis by independent T-tests versus Untreated controls (* = p<0.05, ** = p<0.005, *** = p<0.001). Grey columns representative of pre-treated samples; black bars representative of untreated controls.

**Fig 4:**
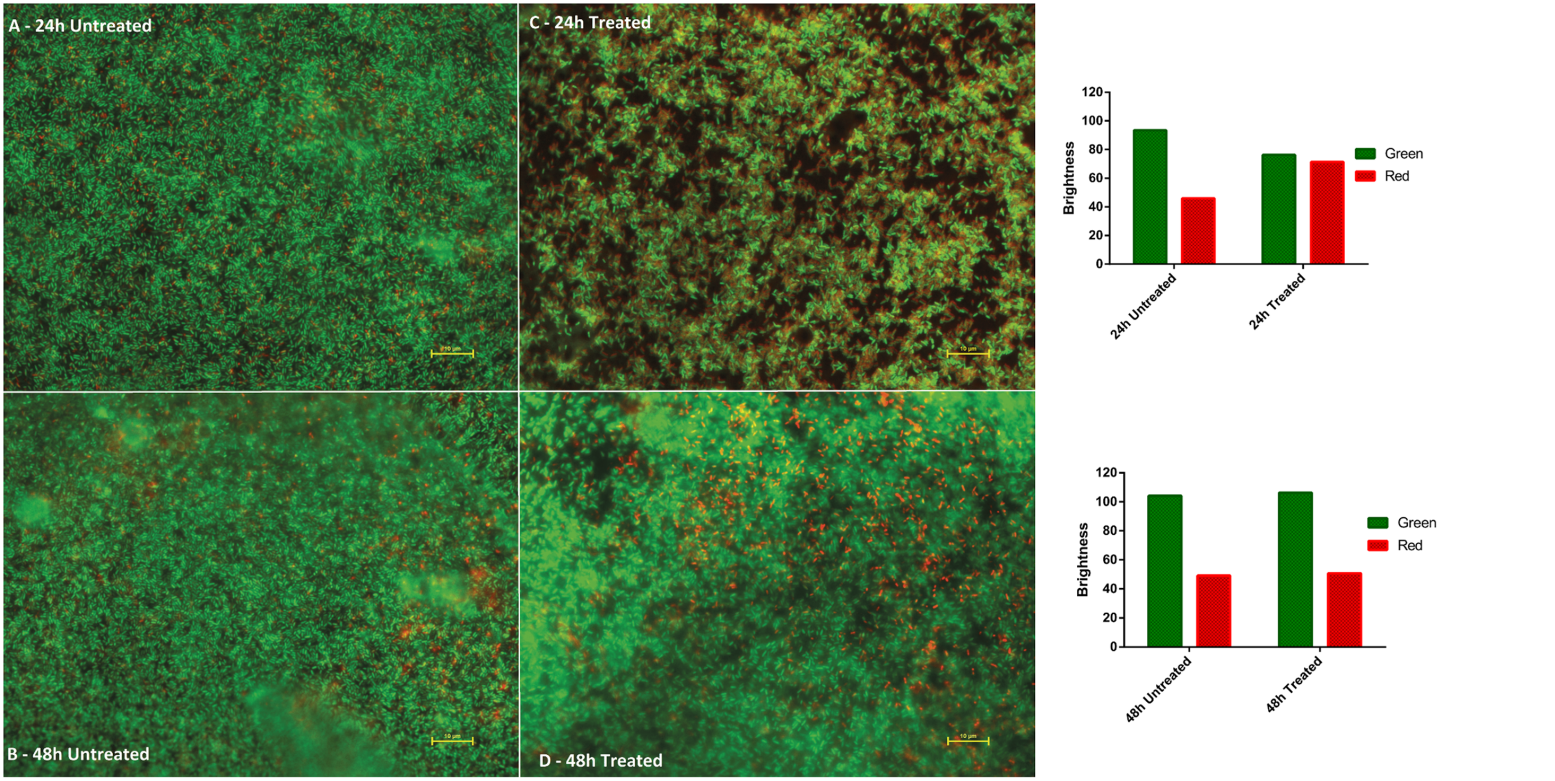
BacLight staining of *P*. *aeruginosa* DSM3227 biofilm at 24 h and 48 h. Live cells appear green and dead/damaged cells appear red. Images A and B show development of extensive biofilm on untreated surfaces. Image C shows biofilm development on GS5 treated surface with a greater proportion of dead/damaged cells. Image D shows GS5 treated surface biofilm at 48 h: biofilm development and cell viability is similar to the untreated control. Images were obtained ×100 magnification (oil immersion) on a Nikon ECLIPSE E400 (Nikon) microscope utilising a dual-band emission filter (450–490 nm/510–560 nm) and NIS-Elements BR (Nikon) software; composite (red/green) images generated using Image J software. Scale bar = 10 μm. Brightness values were generated for each panel (fig 4 a/b/c/d) using ‘imageJ colour histogram analysis’ software which converts RBG pixels to brightness values (V = (R+G+B)/3). These red/green brightness values are presented as bar charts to the right of the micrographs.

Walker *et al*. [9] have demonstrated that biofilm contamination can contribute significantly to outbreaks of healthcare acquired infections. Given the efficacy of GS5 against a range of HAI microbes, we hypothesised that a GS5-treated surface would impede the development of bacterial biofilms. *P*. *aeruginosa* is a well characterised biofilm former [26], and therefore we pre-treated plastic microtitre plate surfaces with GS5 and assessed the development of *P*. *aeruginosa* DSM3227 biofilms. The crystal violet staining method provides a quantitative measure of biofilm development/biomass and somewhat unexpectedly our data revealed that GS5 did not appear to inhibit the development of *P*. *aeruginosa* DSM3227 biofilm in plastic microtiter plates (Fig 3). Having observed that *P*. *aeruginosa* DSM3227 biofilm development was apparently unaffected, we assessed bacterial viability within the biofilms using the well-established BacLight staining method. This analysis suggested that a proportion of the bacterial cells were damaged or rendered non-viable when grown on GS5 treated surfaces, but that, critically, a sufficient number of viable/undamaged cells remained (Fig 4) which, we hypothesise are responsible for subsequent biofilm development.

## Discussion

Only a single published report exists which details the effects of GS5 used as a surface biocide. GS5 is reported to exert its antimicrobial effect via bonding of the silane end of the molecule to surfaces, following which microbes are drawn onto the hydrocarbon chain. The resultant puncturing of cell membranes and denaturation of proteins is proposed as the cause of cell death [20]. As a covalent bond is formed with the surface it is hypothesised that this mode of action is prolonged creating a ‘bactericidal surface’.

When we tested the prolonged activity GS5 exhibited bactericidal activity for 6 days (0.26 log_10_ reduction) whereas the other surface disinfectants tested showed no activity beyond day 0 (Fig 1). In comparison with previous residual testing of the GS5 product by Baxa *et al*. [20], which was completed on fabric swatches rather than on hard surfaces, we observed that residual antimicrobial activity of GS5 was lower (6 days rather than 14 days) [20]. However, the residual antibacterial effect decreased over time to a <1 log_10_ reduction in bacterial numbers, suggesting that GS5 would need regular reapplication and would not be sufficient as a surface disinfectant alone.

GS5 treated surfaces exhibited bactericidal activity which varied in effectiveness between surface type and bacterial species (Table 1). Thus, bacterial species challenged, in addition to surface type/properties, appears to have a significant influence on the performance of the GS5 product. Surface hydrophobicity, charge and roughness have all been reported as important with respect to performance of biocides [12]. Indeed, variations in the response of bacterial species to disinfectants is evident in the literature with disparate log_10_ reductions and widely varying minimum inhibitory concentrations (MICs); biocidal resistance is also evident [20,27]. GS5 is said to not induce resistance in microorganisms as a result of its physical mode of action, reported as membrane disruption and protein denaturation. We noted differences between the results of our current work and data reported by Baxa *et al*. [20] who also tested *S*. *aureus*, *E*. *coli* and *P*. *aeruginosa* on steel and Formica. The work of Baxa *et al*. [20] suggested that GS5 had greater efficacy against *E*. *coli* and *P*. *aeruginosa*, however this observation could be a result of differing surface properties across different types of Steel and Formica used. However, like Baxa *et al*. [20], we have shown that the performance of GS5 against different bacterial species varies considerably, which indicates that the specific type of microbial contaminant will be of greater influence on the effectiveness of GS5, than the actual surface on which it is used.

The ability of HCAI pathogens to adhere, via specific surface proteins to a range of substrates likely to be found in healthcare settings, including polystyrene, has been reported [28]. While biofilms that develop on medical devices such as catheters, chest tubes, prosthetic joints etc. are of concern [29], such medical devices were not the focus of our work. Beyond medical devices, on which biofilms most certainly develop, the contamination of any surface with bacteria in a matrix containing nutrients, will potentially enable development of biofilm. Hospital water systems, from storage to taps, allow biofilm formation and such contamination has been directly linked to adverse health outcomes [9].

Experiments in which plastic surfaces were pre-treated for 10 min with GS5 showed that there was no significant inhibitory effect against *P*. *aeruginosa* biofilm formation (Fig 3). It is well documented that biofilms exhibit increased resistance to antimicrobials and disinfectants, mainly due to the inability of these molecules to penetrate the biofilm [27]. Given that the GS5-treated plate surfaces would be expected to possess antimicrobial activity, we then considered the viability of cells within developing biofilms. Using BacLight, we observed an initial apparent bactericidal effect on *P*. *aeruginosa* DSM3227 cells (Fig 4c) as evidenced by a reduction in biofilm coverage and increased numbers of red stained, damaged, cells at 24 h. This did not translate however, into reduced biofilm formation as measured by crystal violet staining, and indeed later 48 h samples (Fig 4D) showed a well-developed biofilm containing viable cells, similar to that observed in the untreated control (Fig 4B) It is likely, therefore, that residual viable cells maintain the ability to form biofilm and we hypothesise that the cells that are initially damaged by GS5 could actually promote biofilm formation: it has been suggested that dead bacterial cell constituents could comprise a key component of the biofilm or indeed even enhance adhesion and stability of cells, thereby allowing biofilm development [30]. Our data, taken together suggest that GS5 treatment will not significantly inhibit biofilm formation.

## Conclusion

Current NHS Infection control practices require that when choosing disinfectants, a 4–5 Log_10_ reduction is required in viable vegetative bacterial cells within a contact/drying time of 10 min, in addition to a spore reduction of 3 Log_10_ within the same period. When tested directly on a suspension of bacterial cells, GS5 achieved a more than 4 Log_10_ reduction with a 5 min contact time however the residual surface active antimicrobial activity of GS5 was much less, at approximately 1 Log_10_ reduction in bacterial numbers. The surface protective effect of GS5 remained for a further 3–6 days without reapplication of the product, however we noted a diminution of the measured Log_10_ reductions over time to a level which was much lower than that required for use in infection control.

Bacteria can form biofilm on surfaces allowing prolonged survival and increased resistance to biocides. Considering the GS5 mode of action we hypothesised a regime where GS5 could be utilised to prevent biofilm formation on surfaces subsequently reducing risk of infection. However GS5 has been shown to possess limited anti-biofilm properties as biofilm production is not impeded on GS5 coated surfaces.

Within the NHS, certain disinfectants (for example, DifficilS) routinely achieve 4 Log_10_ reductions in both vegetative cell and spore numbers within 3–5 min however control of infection is only achievable in practice by using these products in intensive cleaning up to twice daily in a rolling programme of disinfection. Thus, on the basis of the data generated in this work, it appears unlikely, despite modest reductions in bacterial cell viability and evidence for a short lived residual effect, that GS5 would replace current infection control products such as DifficilS or Actichlor in reducing the transmission of HAI pathogens within hospitals and care settings.

## Supporting information

S1 Table Supporting dataset of plate count & A570 data. (XLSX)
